# Dynapenic abdominal obesity and activities of daily living disability among older adults residing in low- and middle-income countries

**DOI:** 10.1007/s40520-024-02864-x

**Published:** 2024-10-26

**Authors:** Lee Smith, Guillermo F. López Sánchez, Pinar Soysal, Karel Kostev, Louis Jacob, Nicola Veronese, Mark A. Tully, Laurie Butler, Yvonne Barnett, Damiano Pizzol, Jae Il Shin, Ai Koyanagi

**Affiliations:** 1 Centre for Health Performance and Wellbeing, Anglia Ruskin University, Cambridge, UK; 2 Division of Preventive Medicine and Public Health, Department of Public Health Sciences, School of Medicine, University of Murcia, Murcia, Spain; 3 Department of Geriatric Medicine, Faculty of Medicine, Bezmialem Vakif University, Istanbul, Turkey; 4 University Clinic of Marburg, Marburg, Germany; 5 Research and Development Unit, Parc Sanitari Sant Joan de Déu, Dr. Antoni Pujadas, Sant Boi de Llobregat, Barcelona, Spain; 6 AP-HP, Université Paris Cité, Lariboisière-Fernand Widal Hospital, Department of Physical Medicine and Rehabilitation, Paris, France; 7 Université Paris Cité, Inserm U1153, Epidemiology of Ageing and Neurodegenerative Diseases (EpiAgeing), Paris, France; 8 Geriatric Unit, Department of Internal Medicine and Geriatrics, University of Palermo, Palermo, Italy; 9 School of Medicine, Ulster University, Londonderry, UK; 10 Italian Agency for Development Cooperation, Khartoum, Sudan; 11 Department of Pediatrics, Yonsei University College of Medicine, Seoul, Republic of Korea; 12 Severance Underwood Meta-Research Center, Institute of Convergence Science, Yonsei University, Seoul, Republic of Korea

**Keywords:** Dynapenic abdominal obesity, ADL disability, Older adults, Low- and middle-income countries

## Abstract

**Background:**

Dynapenic abdominal obesity (DAO) may be associated with an increased risk of disability. However, to date, this has not been investigated in low- and middle-income countries (LMICs), while the mediators are largely unknown.

**Aims:**

Therefore, we aimed to investigate the association between DAO and activities of daily living (ADL) disability, and to identify potential mediators among older adults from six LMICs.

**Methods:**

Cross-sectional, nationally representative data from the WHO Study on global AGEing and adult health were analyzed. Data on 20,198 adults aged ≥ 60 years were analyzed [mean (SD) age 69.3 (13.1) years; 54.1% females]. Dynapenia was defined as handgrip strength of < 26 kg for men and < 16 kg for women. Abdominal obesity was defined as waist circumference of > 88 cm for women and > 102 cm for men. DAO was defined as having both dynapenia and abdominal obesity. Disability was defined as severe or extreme difficulty in conducting at least one of six types of ADL. Multivariable logistic regression and mediation analysis were conducted.

**Results:**

Compared to no dynapenia and no abdominal obesity, DAO was significantly associated with 2.08 (95%CI = 1.37–3.17) times higher odds for ADL disability Mediation analysis showed that diabetes (mediated percentage 4.7%), hypertension (7.2%), and angina (7.7%) were significant mediators in the association between DAO and ADL disability.

**Conclusions:**

DAO was associated with increased odds for ADL disability among older adults from LMICs. Future longitudinal studies are warranted to assess temporal associations, and whether addressing or preventing DAO can impact on future occurrence of disability.

## Introduction

Activities of daily living (ADL) collectively describe fundamental skills required to independently care for oneself, such as eating, bathing, and mobility [1, 2], and ADL is an indicator of one’s functional status. The inability to perform ADL results in the dependence on other individuals and/or mechanical devices, while the inability to accomplish essential ADLs may lead to unsafe conditions and poor quality of life [3]. Furthermore, people with disability are known to be at higher risk for premature mortality [4]. It is thus of prime importance to identify risk factors of ADL disability to inform targeted intervention. This is particularly so in the context of older adults in low- and middle-income countries (LMICs) as limitations in ADL increase with age, while the speed of ageing in LMICs is outpacing that of high-income countries. Indeed, the United Nation estimates that two-thirds of the world’s population aged 60 years and over will be living in LMICs by 2050 [5].

One potentially important but understudied risk factor of ADL disability is dynapenic abdominal obesity (DAO; i.e., impairment in muscle strength and high waist circumference) [6]. DAO can theoretically increase risk for disability through several mechanisms. First, DAO may increase risk for diabetes owing to, for example, muscle strength being inversely associated with insulin resistance [7–9], and an increase in secretion of non-esterified fatty acids and adipocytokines associated with central obesity [7]. In turn, chronic complications of diabetes (e.g., chronic vascular disease, neuropathy) can result in disability [10]. Second, DAO increases risk for hypertension, stroke, and angina due to, for instance, a poor inflammatory profile, and these conditions can lead to disability via their symptoms per se or the end result (e.g., congestive heart failure, renal disease) in the case of hypertension [11]. Third, DAO may increase risk for falls via decreased postural stability due to abdominal obesity, and inability to correct posture due to impaired muscular systems [12, 13], while injury caused by falls may lead to ADL disability. Finally, poor inflammatory profiles in DAO may increases risk for cognitive impairment, which can lead to limitations in ADL via a reduction in executive function, which is instrumental in the organization and regulation of mental processes and voluntary behavior [14].

Despite the numerous ways in which DAO may potentially increase risk for disability, to date, there are very few studies that have investigated the association between DAO and disability. For example, in one study with 9 years of follow-up including 370 men and 476 women aged between 65 and 95 years from Italy, those with DAO presented more than a twofold increase in risk of worsening disability (OR = 2.10; 95%CI = 1.14–3.88) compared to those without dynapenia and abdominal obesity [15]. Furthermore, in another Italian study including 93 men and 169 women aged between 66 and 78 years with over 5.5 years of follow-up, DAO more than trebled the risk (HR:3.39,95%CI:1.91–6.02) of disability worsening (vs. no dynapenia and no abdominal obesity) [16]. Additionally, in one UK study including 3,723 participants aged 50 years and older with 8-years of follow-up, it was observed that the estimated change over time in ADL disability was significantly higher for participants with DAO compared with those with neither dynapenia or abdominal obesity [17]. Similar findings were found within the same dataset when utilizing instrumental ADLs as the outcome [18]. To the best of the authors’ knowledge, no other studies exist on the association between DAO and disability. Given that all previous studies were conducted in high-income countries with relatively small sample sizes, it is clear that more research from other settings with large sample size are needed to assess whether study results can be replicated. Furthermore, there is the need to understand the potential mediators in this association to shed light on the mechanisms that may underlie this association.

Given this background, the aim of the present study was to investigate the association between DAO and ADL disability, and to what extent diabetes, hypertension, stroke, angina, injurious fall, and cognition may mediate this association in a sample of 20,198 adults aged ≥ 60 years from six LMICs.

## Methods

### The survey

The present study utilized data from the Study on Global AGEing and Adult Health (SAGE). The survey was implemented between 2007 and 2010 across six countries (China, Ghana, India, Mexico, Russia, and South Africa). When the survey was implemented and according to the World Bank classification, Ghana was a low-income country, and China and India were lower middle-income countries. However, in 2010 China transitioned to an upper middle-income country. The other four countries were upper middle-income countries. The methodology of the survey has previously been published [19]. In brief, a multistage clustered sampling design method was used to acquire nationally representative samples. Those aged ≥ 18 years were included and those aged ≥ 50 years were oversampled. Face to face interviews were carried out by trained interviewers utilizing a standardized questionnaire. Standardized translation protocols were carried out for comparability between countries. Response rates to the surveys were as follows: China 93%; Ghana 81%; India 68%; Mexico 53%; Russia 83%; and South Africa 75%. To adjust for the population structure, as reported by the United Nations Statistical Division, sampling weights were developed. Ethical approval was acquired from the WHO Ethical Review Committee and local ethics research review boards. All participants provided written informed consent prior to participation.

### ADL disability

ADL disability was assessed with six questions on the level of difficulty in conducting standard basic ADL in the past 30 days (washing whole body, getting dressed, moving around inside home, eating, getting up from lying down, and using the toilet) [20]. Those who answered severe or extreme/cannot do to any of the six questions were considered to have ADL disability [21].

### Dynapenia, abdominal obesity, and dynapenic abdominal obesity

Handgrip strength was measured using a Smedley Hand Dynamometer (Scandidact Aps, Denmark). Dynapenia was defined as < 26 kg for men and < 16 kg for women [22], using the average value of the two handgrip measurements of the dominant hand. Waist circumference was measured at the midpoint between the lower margin of the least palpable rib and the top of the iliac crest keeping the measuring tape parallel to the floor. Abdominal obesity was defined as a waist circumference of > 88 cm for women and > 102 for men [23]. Participants were divided into four groups according to dynapenia and abdominal obesity status: No dynapenia and no abdominal obesity, dynapenia alone, abdominal obesity alone, and dynapenia and abdominal obesity (i.e., DAO).

### Mediators

The potential mediators (i.e., diabetes, hypertension, stroke, angina, injurious fall, cognition) were selected based on past literature suggesting that they can be the result of dynapenic abdominal obesity, and be the cause of disability [12, 24–26]. Diabetes and stroke were solely based on lifetime self-reported diagnosis, while angina referred to lifetime self-reported diagnosis and/or diagnosis based on the Rose questionnaire [27]. Hypertension was defined as having at least one of the following: systolic blood pressure ≥ 140 mmHg; diastolic blood pressure ≥ 90 mmHg; or self-reported diagnosis. For injurious falls, the participant was first asked “In the past 12 months, have you had any other event (other than a road traffic accident) where you suffered from bodily injury?” Those who answered affirmatively were prompted to the next question “What was the cause of the injury?” If the respondent answered “Fall”, then he or she was considered to have had a fall-related injury in the past year. Cognition was assessed with two questions on difficulty concentrating/remembering things or learning a new task in the past 30 days, with five answer options (none to extreme). Factor analysis with polychoric correlations was conducted to create a scale ranging from 0 to 100 with higher values representing worse cognition [28, 29].

### Control variables

The selection of the control variables was based on past literature [15, 17], and included age, sex, country-wise wealth quintiles based on income, highest level of education achieved (≤ primary, secondary, tertiary), smoking (never, current, past), physical activity, and alcohol consumption. Levels of physical activity were assessed with the Global Physical Activity Questionnaire and were classified as low, moderate, and high based on conventional cut-offs [30]. Consumers of at least four (females) or five drinks (males) of any alcoholic beverage per day on at least one day in the past week were considered ‘heavy’ drinkers. Those who had ever consumed alcohol but were not heavy drinkers were categorized as ‘non-heavy’ drinkers [31].

### Statistical analysis

The statistical analysis was done with Stata 14.2 (Stata Corp LP, College station, Texas). The analysis was restricted to those aged ≥ 60 years. The difference in sample characteristics by ADL disability status was tested by Chi-squared tests, except for age (Student’s *t*-tests). Multivariable logistic regression analysis was conducted to assess the association between.

the four-category variable on dynapenia, abdominal obesity, or both (exposure) and ADL disability (outcome), with no dynapenia and no abdominal obesity being the reference category. In order to assess whether the magnitude of the association differs by sex, we conducted interaction analysis by including the product term of the variable on dynapenia/abdominal obesity status and sex in the model. Since preliminary analysis showed that there is no significant interaction, analyses were not stratified by sex. Finally, mediation analysis was conducted to gain an understanding of the extent to which various factors (i.e., diabetes, hypertension, stroke, angina, injurious fall, cognition) may explain the association between dynapenic abdominal obesity and ADL disability. The exposure variable for this analysis was a dichotomized variable on presence or absence of DAO. We used the *khb* (Karlson Holm Breen) command in Stata [32] for the mediation analysis. This method can be applied in logistic regression models and decomposes the total effect (i.e., unadjusted for the mediator) of a variable into direct (i.e., effect of dynapenic abdominal obesity on ADL disability adjusted for the mediator) and indirect effects (i.e., mediational effect). Using the *khb* command, the percentage of the main association explained by the mediator can also be calculated (mediated percentage). Each potential mediator was included in the model individually, with the exception of the model where all potential mediators were included in the model simultaneously. All regression analyses including the mediation analysis were adjusted for age, sex, wealth, education, smoking, physical activity, alcohol consumption, and country. Adjustment for country was done by including dummy variables for each country in the model as in previous SAGE publications [33, 34]. The sample weighting and the complex study design were considered in all analyses. Results from the regression analyses are presented as odds ratios (ORs) with 95% confidence intervals (CIs). The level of statistical significance was set at *P* < 0.05.

## Results

**Fig. 1:**
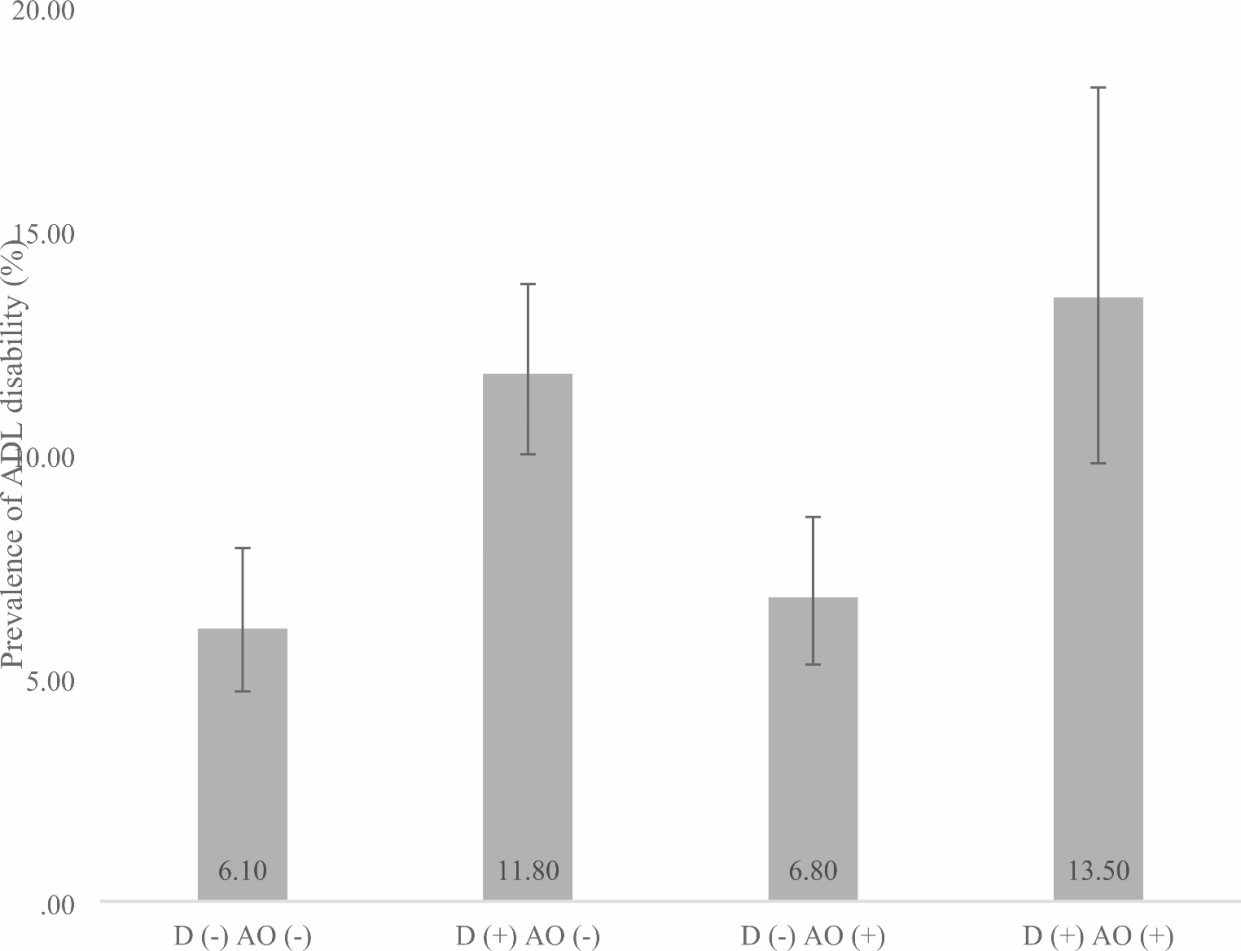
Prevalence of ADL disability by dynapenia and abdominal obesity status

Data on 20,198 adults aged ≥ 60 years were analyzed (China *n* = 7474; Ghana *n* = 2616; India *n* = 3621; Mexico *n* = 1879; Russia *n* = 2465; South Africa *n* = 2143). The prevalence of ADL disability was 10.1%, while the prevalence of dynapenia alone, abdominal obesity alone, and DAO were 33.3%, 15.8%, and 7.7%, respectively. The sample characteristics are shown in Table 1. The mean (SD) age of the sample was 69.3 (13.1) years and 54.1% were females. People with ADL disability were significantly more likely to be older, be females, and have low levels of wealth, education, and physical activity, while they were less likely to consume alcohol. The prevalence of ADL disability was particularly high among those with DAO (13.5%) (Fig. 1). After adjustment for potential confounders, compared to no dynapenia and no abdominal obesity, DAO was significantly associated with 2.08 (95%CI = 1.37–3.17) times higher odds for ADL disability (Table 2). In contrast, dynapenia alone and abdominal obesity alone were not significantly associated with ADL disability. Mediation analysis showed that diabetes (mediated percentage 4.7%), hypertension (7.2%), and angina (7.7%) were significant mediators in the association between DAO and ADL disability, while stroke, injurious falls, and cognition were not significant mediators (Table 3). All potential mediators collectively explained 19.4% of the association between DAO and ADL disability.

**Table 1:** Sample characteristics (overall and by ADL disability)

			ADL disability		
Characteristic		Overall	No	Yes	P-value^a^
Age (years)	Mean (SD)	69.3 (13.1)	68.9 (12.5)	73.1 (15.0)	< 0.001
Sex	Female	54.1	53.0	62.8	< 0.001
	Male	45.9	47.0	37.2	
Wealth	Poorest	20.1	19.7	24.5	0.001
	Poorer	20.2	20.0	21.6	
	Middle	20.5	20.0	23.6	
	Richer	18.8	19.1	16.4	
	Richest	20.3	21.2	13.8	
Education	≤Primary	63.9	62.9	70.6	0.003
	Secodary	29.8	30.5	25.9	
	Tertiary	6.3	6.6	3.5	
Smoking	Never	60.6	60.8	59.3	0.155
	Current	31.6	31.7	30.5	
	Past	7.8	7.5	10.2	
Physical activity	High	40.0	42.0	23.4	< 0.001
	Moderate	24.8	25.5	17.2	
	Low	35.2	32.5	59.4	
Alcohol consumption	Never	67.8	67.1	74.0	0.001
	Non-heavy	29.2	29.6	25.2	
	Heavy	3.0	3.3	0.8	

*Abbreviation* SD Standard deviation

Data are % unless otherwise stated

^a^ P-value was based on Chi-squared tests except for age (Student’s *t*-test)

**Table 2:** Association between Dynapenia, abdominal obesity, or both and ADL disability (outcome) estimated by multivariable logistic regression

Characteristic		OR	95%CI	*P*-value
Dynapenia & abdominal	D (-) AO (-)	1.00		
obesity status	D (+) AO (-)	1.31	[0.90,1.90]	0.159
	D (-) AO (+)	0.91	[0.58,1.42]	0.675
	D (+) AO (+)	2.08	[1.37,3.17]	0.001
Age (years)		1.05	[1.03,1.06]	< 0.001
Sex	Female	1.00		
	Male	0.69	[0.51,0.95]	0.023
Wealth	Poorest	1.00		
	Poorer	0.96	[0.68,1.35]	0.809
	Middle	1.05	[0.74,1.49]	0.780
	Richer	0.87	[0.61,1.24]	0.431
	Richest	0.54	[0.36,0.80]	0.002
Education	≤Primary	1.00		
	Secondary	0.79	[0.54,1.14]	0.210
	≥Tertiary	0.54	[0.27,1.07]	0.078
Smoking	Never	1.00		
	Current	0.97	[0.67,1.40]	0.860
	Past	1.45	[0.95,2.20]	0.083
Physical activity	High	1.00		
	Moderate	1.17	[0.88,1.54]	0.279
	Low	2.43	[1.75,3.36]	< 0.001
Alcohol consumption	Never	1.00		
	Non-heavy	0.99	[0.65,1.50]	0.961
	Heavy	0.73	[0.26,2.04]	0.545

*Abbreviation* OR Odds ratio; CI Confidence interval; D Dynapenia; AO Abdominal obesity

Model is adjusted for all variables in the Table and country

**Table 3:** Mediators in the association between dynapenic abdominal obesity and ADL disability

Mediator	Effect	OR [95%CI]	*P*-value	%Mediated^a^
Diabetes	Total	1.96 [1.33,2.88]	0.001	4.7
	Direct	1.90 [1.28,2.80]	0.001	
	Indirect	1.03 [1.004,1.06]	0.026	
Hypertension	Total	1.94 [1.31,2.86]	0.001	7.2
	Direct	1.85 [1.24,2.75]	0.002	
	Indirect	1.05 [1.004,1.10]	0.035	
Stroke	Total	1.96 [1.33,2.91]	0.001	NA
	Direct	1.93 [1.31,2.86]	0.001	
	Indirect	1.02 [1.00,1.04]	0.132	
Angina	Total	1.93 [1.31,2.85]	0.001	7.7
	Direct	1.84 [1.24,2.73]	0.003	
	Indirect	1.05 [1.01,1.10]	0.014	
Injurious fall	Total	1.92 [1.30,2.81]	0.001	NA
	Direct	1.92 [1.30,2.82]	0.001	
	Indirect	1.00 [0.98,1.01]	0.953	
Cognition	Total	2.08 [1.39,3.11]	< 0.001	NA
	Direct	2.00 [1.34,3.00]	0.001	
	Indirect	1.04 [0.96,1.12]	0.357	
All mediators	Total	2.08 [1.36,3.16]	0.001	19.4
	Direct	1.80 [1.18,2.76]	0.007	
	Indirect	1.15 [1.04,1.28]	0.007	

*Abbreviation* OR Odds ratio; CI Confidence interval

Models are adjusted for age, sex, wealth, education, smoking, physical activity, alcohol consumption, and country

^a^ % Mediated was only calculated in the presence of a significant indirect effect (i.e., *P* < 0.05)

Abbreviation: D Dynapenia; AO Abdominal obesity

Bars denote 95% confidence interval

## Discussion

In our large study including more than 20,000 older adults from LMICs, after adjustment for potential confounders, compared to no dynapenia and no abdominal obesity, DAO more than doubled the odds for ADL disability (OR = 2.08; 95%CI = 1.37–3.17), while dynapenia alone and abdominal obesity alone were not significantly associated with ADL disability. Diabetes (mediated percentage 4.7%), hypertension (7.2%), and angina (7.7%) were identified as significant mediators in the association between DAO and ADL disability, but these factors only explained a relatively small proportion of the association between DAO and ADL disability. To the best of our knowledge, this is the first study on this topic from LMICs, while it is also the first to examine the potential mediators of the DAO-ADL disability association.

Findings from the present study both support and add to the previous literature. They support previous literature from high-income countries through further confirming that an association exists between DAO and disability [15–17] in a large representative sample of older adults from multiple LMICs. Furthermore, our study showed that diabetes, hypertension, and angina are potential mediators in the association between DAO and ADL disability but that their influence in this association may be relatively small.

As previously discussed, DAO may increase risk of diabetes, hypertension, and angina via factors such as insulin resistance and inflammation, and these conditions are known to increase risk for disability [35]. However, the fact that the influence of these factors was minimal suggests that there are other important mechanisms that underlie the association between DAO and disability. For instance, this may be explained by the conceptual model of disability proposed by Rivera and colleagues [36]. This proposes that six domains are likely to explain an increased risk in ADLs (disability), including: central nervous system, peripheral nervous system, muscular system, osteoarticular system (bones and joints), perceptual system, and energy production. Indeed, the excess of macronutrients in adipose tissues stimulates the tissue to release inflammatory mediators such as tumor necrosis factor α and interleukin 6, and reduces production of adiponectin, predisposing to a pro-inflammatory state and oxidative stress as well as increasing muscle catabolic activity [37]. Moreover, abdominal adiposity increases the risk of intermuscular and intramuscular fat infiltration altering the muscular anatomy and consequently impairing its function. These changes impact the functioning of the peripheral nervous system and muscular system [17]. Importantly, when the neuromuscular system is impaired, it may result in a higher risk of falls [38] and in difficulties dealing with an overload in the osteoarticular system caused by abdominal obesity and consequently results in limitations in ADLs [17].

The large representative sample of older adults from six LMICs, and the investigation of potential mediating variables in the DAO-ADL relationship, are clear strengths of the present study. However, findings must be interpreted in light of the study limitations. First, the study was cross-sectional in nature, and thus, the direction of the observed association cannot be confirmed. It is also possible that limitations in ADL increase risk for DAO through, for example, a reduction in energy expenditure resulting in an increase in adiposity and a reduction in muscle strength. However, previous longitudinal studies have observed that DAO is longitudinally associated with worsening disability [17]. Second, some variables were self-reported, potentially introducing recall and social desirability bias into the findings. Finally, given that the institutionalized were excluded from the survey, our study results cannot be generalized to this population which could have higher prevalence of DAO and limitations in ADLs.

In conclusion, people with DAO were at more than two times higher odds for disability, compared with people with no dynapenia or abdominal obesity. Interestingly, in our study, having dynapenia alone or abdominal obesity alone was not significantly associated with disability, highlighting the importance of the co-existence of these conditions in terms of disability risk. Given that the identified potential mediators only explained a small proportion of the association between DAO and ADL disability, future studies that further explore the underlying mechanisms are warranted. Finally, future longitudinal studies should assess whether addressing DAO can lead to a reduction in the future onset of ADL disability among older adults in LMICs.

## Data Availability

No datasets were generated or analysed during the current study.
